# Relating Alpha Power and Phase to Population Firing and Hemodynamic Activity Using a Thalamo-cortical Neural Mass Model

**DOI:** 10.1371/journal.pcbi.1004352

**Published:** 2015-09-03

**Authors:** Robert Becker, Stuart Knock, Petra Ritter, Viktor Jirsa

**Affiliations:** 1 Functional Brain Mapping Lab, University of Geneva, Geneva, Switzerland; 2 Institut de Neurosciences des Systèmes, Aix Marseille Université, Marseille, France; 3 Minerva Research Group BrainModes, Max Planck Institute for Human Cognitive and Brain Sciences, Leipzig, Germany; 4 Dept. Neurology, Charité & Bernstein Center for Computational Neuroscience—University Medicine, Berlin, Germany; 5 Berlin School of Mind and Brain & Mind and Brain Institute, Humboldt University, Berlin, Germany; 6 Inserm, UMR 1106, Aix Marseille Université, Marseille, France; Brain and Spine Institute (ICM), France

## Abstract

Oscillations are ubiquitous phenomena in the animal and human brain. Among them, the alpha rhythm in human EEG is one of the most prominent examples. However, its precise mechanisms of generation are still poorly understood. It was mainly this lack of knowledge that motivated a number of simultaneous electroencephalography (EEG) – functional magnetic resonance imaging (fMRI) studies. This approach revealed how oscillatory neuronal signatures such as the alpha rhythm are paralleled by changes of the blood oxygenation level dependent (BOLD) signal. Several such studies revealed a negative correlation between the alpha rhythm and the hemodynamic BOLD signal in visual cortex and a positive correlation in the thalamus. In this study we explore the potential generative mechanisms that lead to those observations. We use a bursting capable Stefanescu-Jirsa 3D (SJ3D) neural-mass model that reproduces a wide repertoire of prominent features of local neuronal-population dynamics. We construct a thalamo-cortical network of coupled SJ3D nodes considering excitatory and inhibitory directed connections. The model suggests that an inverse correlation between cortical multi-unit activity, i.e. the firing of neuronal populations, and narrow band local field potential oscillations in the alpha band underlies the empirically observed negative correlation between alpha-rhythm power and fMRI signal in visual cortex. Furthermore the model suggests that the interplay between tonic and bursting mode in thalamus and cortex is critical for this relation. This demonstrates how biophysically meaningful modelling can generate precise and testable hypotheses about the underpinnings of large-scale neuroimaging signals.

## Author Summary

In this article, we show how a large-scale neuronal model involving a thalamo-cortical circuit can reproduce findings from human and animal brain oscillatory activity and how this can help understanding the mechanisms, which generate this activity. Amongst the brain rhythms, the alpha rhythm (8-12Hz) is the most prominent, having its most pronounced expression when the brain is at rest. The mechanisms underlying its generation are not fully understood. Using computational modelling techniques, we demonstrate how the alpha rhythm may emerge from local and network interactions and explain empirical results, such as why the energy consumption drops when electric brain activity—as indicated by the amplitude of oscillations—goes up.

## Introduction

The mechanisms underlying human alpha rhythm generation are still insufficiently understood. One reason is that direct access to human brain activity is limited. Thus, mainly animal studies guide neuroscientists in their quest to reveal these mechanisms. Seminal studies employed dog animal models to explore the cellular substrates of the alpha rhythm [1]. A debated issue is whether alpha oscillations are an emergent property of thalamo-cortical connections or yielded by cortical networks independent of thalamic control. Lopes da Silva et al. were the first to show that a) there was considerable but not absolute coherence between thalamic and cortical alpha oscillations [1] and b) that by performing a ‘virtual deafferentiation’ as achieved by partial coherence analysis, there was still considerable coherence within the cortex independent of a thalamic pace maker [2]. Another finding possibly related to generation mechanisms of alpha activity was that sleep spindles oscillating at frequencies partially overlapping with the alpha band are founded on the capability of thalamic nuclei to switch between tonic and phasic activity, the latter sometimes also referred to as bursting mode. This behaviour is caused by an interplay between neuronal populations in the thalamic relay nucleus and the inhibitory reticular nucleus [3]. More recently, it has been shown in-vitro that a bursting mode mechanism might also be relevant for the generation and propagation of alpha oscillations (e.g. [4,5]). Complementary to this conceptual finding, another study in monkeys has recently demonstrated that the alpha (or mu) amplitude in somato–sensory cortex is not only inversely correlated to sensory discrimination performance but also to firing rate of the involved neuronal ensemble [6]. Furthermore, this study also demonstrated a robust coupling between alpha-phase and firing rate, supporting the idea of high-frequency bursting activity being related to rhythmic alpha activity. Taken together, these findings point to a critical role for the bursting behaviour of neurons in the generation of human alpha activity. In how far this might be achieved locally or as a feature of a thalamo-cortical network is not clear, however.

In humans, functional magnetic resonance imaging (fMRI)–one of the prevalent non-invasive brain imaging methods–has been combined with electro–encephalography (EEG, for a review see [7] to further explore the alpha rhythm. Numerous studies revealed a negative correlation between the strength of the classical posterior alpha rhythm derived by EEG and the blood oxygen level dependent (BOLD) signal—predominantly in posterior, i.e. mainly visual, cortical regions [8–11], but see also [1,12]. A mechanistic biophysical understanding of this negative relation between rhythm strength and BOLD signal amplitude, however, has been lacking.

**Fig 1:**
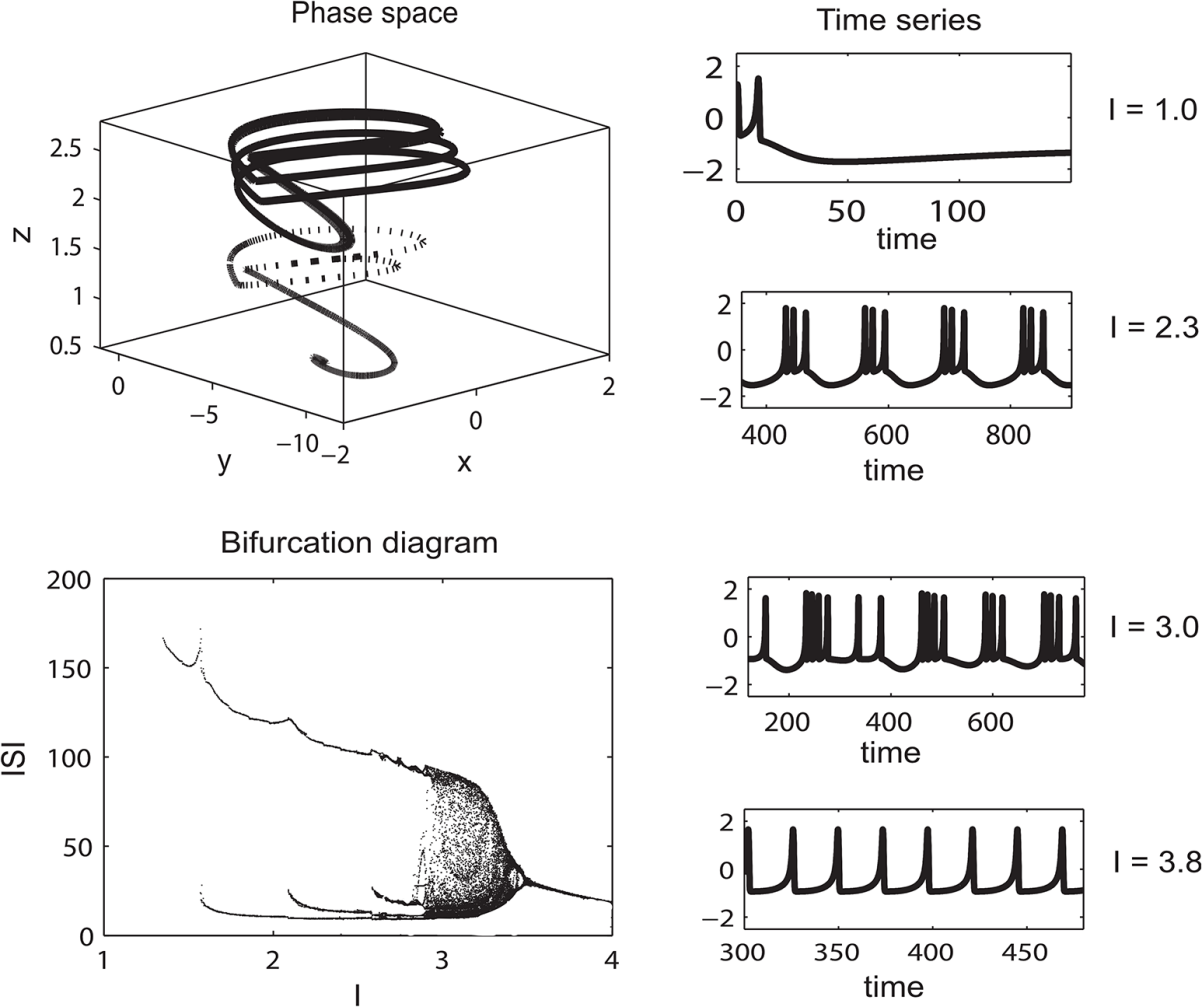
Example of the dynamic range captured by the neuronal activity of the HR model. On the top left, a typical trajectory in the phase space is shown. Bottom left shows the bifurcation diagram of interspiking intervals for different ranges of the parameter I. On the right, corresponding time series are shown. Depending on the parameter I, the system shows a wide range of behaviours, from regular spiking to bursting to chaotic regimes and fixed point behaviour. Adapted from [13](parameter values a = 1; b = 3; c = 1; d = 5; s = 4; r = 0.006; x0 = 21.6).

**Fig 2:**
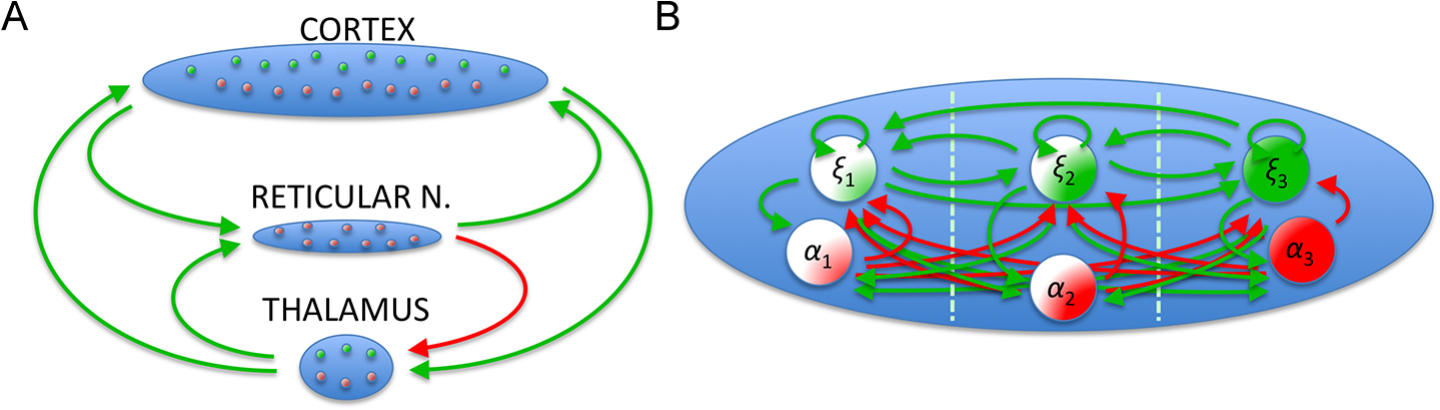
*A*. The general large-scale connectivity of the thalamo-cortical model is shown across the nodes. The model comprises the three network nodes (cortex, reticular nucleus and thalamus) known to be major processing units of the visual system. The model accounts for inhibitory and excitatory connections (here, red is inhibitory, green is excitatory) and their directionality as well as anatomical distances. ***B*.** The intrinsic connectivity across the 3 modes of the SJ3D within a single node is shown (with *ξ* being the main state variable for excitatory activity and *α* for inhibitory activity).

In the present study we present a generative thalamo-cortical model that attempts to bridge the gap between invasive findings in animals and non-invasive multimodal imaging in humans. Specifically, we provide a model that integrates the inverse relationship between the alpha-rhythm power and the fMRI-BOLD signal in humans and the negative relationship between alpha rhythm and firing rate found invasively in animals. Our thalamo-cortical model consists of coupled Stefanescu-Jirsa 3D (SJ3D) nodes, i.e. connected mean field models derived from populations of spike-burst neurons with distributed parameters [1,13]. Derived from Hindmarsh-Rose single neurons [2,14], see also Fig 1), the neural activity generated by these SJ3D nodes accounts for a wide repertoire of dynamical regimes observable in empirical electrophysiological data. In particular, the SJ3D model is the only mean field model accounting for bursting activity, hence the prime candidate for modelling of the hypothesized spike-burst patterns of thalamic nuclei. Our working hypothesis is that a set of appropriately coupled large-scale spike-burst nodes can account for the above-mentioned features of human alpha rhythm activity or corresponding activity in animals. We define the nodes of our neuronal network model by choosing the anatomical structures that are assumed to be most relevant for the generation of the posterior alpha rhythm: a node representing the reticular nucleus, a node representing a relay nucleus of the thalamus e.g. such as pulvinar or lateral geniculate body and a node representing the visual cortex. We will further refer to them as reticular, thalamic and cortical node. Each node is represented by a SJ3D model that provides a reduced description of the dynamics of a meso-scale network of coupled inhibitory and excitatory neurons. The individual nodes are coupled through a biologically plausible connectivity skeleton accounting for physiological time delays, the quality of interaction, i.e. inhibition versus excitation, and the direction of information flow (for a schematic description cf. Fig 2, for exact values see Table 1). Based on the model output we approximate multi-unit activity, local field potentials, alpha-band power as well as the hemodynamic BOLD response of the relevant nodes in our network. We then vary the parameter of global connectivity, i.e. the connectivity-scaling factor (CSF) in the model systematically. Subsequently, we analyse its effect on the model output and on the following features of the alpha rhythm: a) spectral power and spectral coherence; b) relationship between amplitude and phase of alpha oscillations and multi-unit activity; c) relationship between alpha amplitude and the predicted BOLD-fMRI response generated by a convolution model. Finally we compare the model predictions to empirical findings and discuss these results in the context of existing literature.

## Results

### Output of SJ3D model

**Fig 3:**
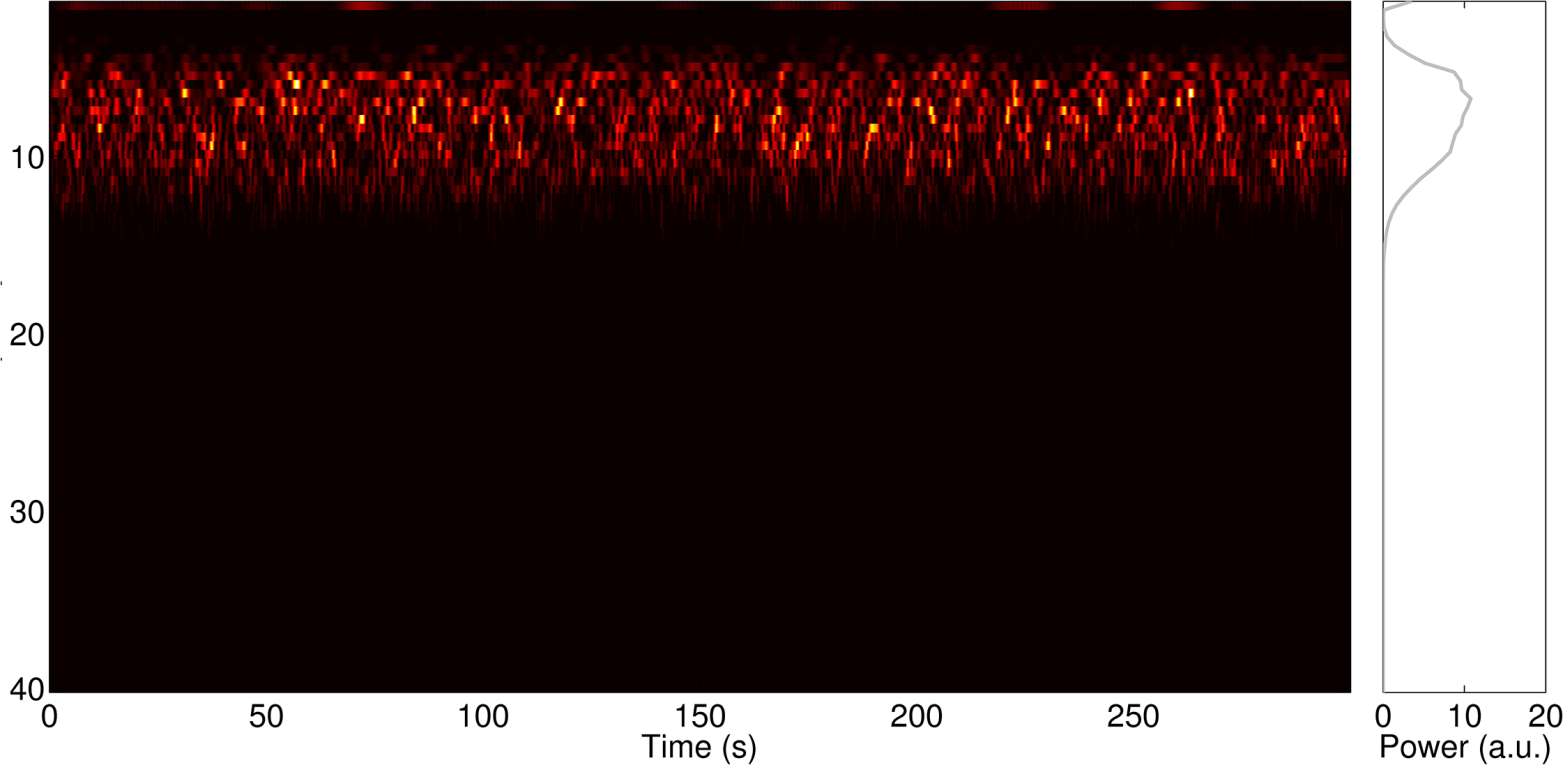
For a CSF of 0.6, the cortical node yields neural oscillations in a narrow frequency band and with fluctuating power over time—similar to the alpha rhythm observable in empirical human EEG data.

**Fig 4:**
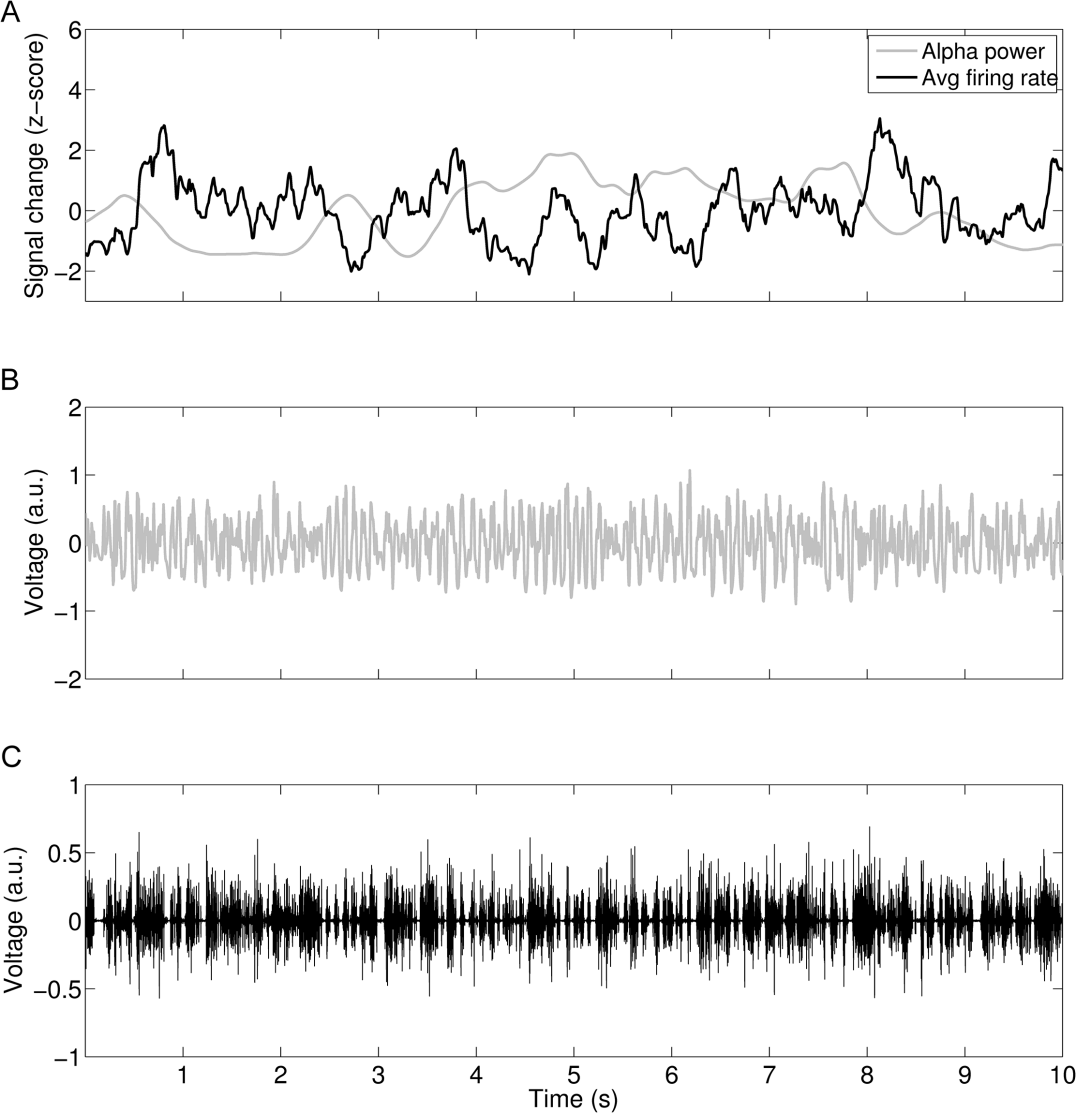
*A*. In grey the fluctuating 8–12 Hz alpha power that has been calculated from the summed excitatory activity of all three modes of the SJ3D model. Alpha-power is moderately, yet systematically, inversely correlated to the mean firing rate (black) of the cortical node. ***B*.** The LFP time series show spontaneous fluctuations within the alpha-band. Low-amplitude alpha-oscillatory modes are accompanied by higher frequency activity. ***C*.** The MUA of the cortical node shows rhythmic bursting associated with an increase in alpha-band power (values shown for CSF = 0.6).

Using the specified parameters and a moderate connectivity strength with a connectivity strength factor (CSF) of 0.6, the cortical node yielded band-limited oscillations around 10 Hz for the simulated local field potential (see section ‘Methods‘ for detailed description of how we derived an approximated LFP from the model output). The amplitude of these oscillations fluctuates as visible in the time-frequency analysis of the local field potential (LFP, Fig 3, raw time series in Fig 4, middle panel). Fig 4 shows a zoom-in for a representative time window of 10-s duration, depicting the alpha-power time course (top panel, grey), the LFP time course (middle panel, grey) as well as the time course of the multi-unit activity (MUA, bottom panel, black, see section ‘Methods‘ for derivation of MUA). Additionally, an estimation of the total firing rate at the cortical node is overlaid on the alpha power (top panel, black). The alpha-power fluctuations are accompanied by rhythmic bursting in the higher frequency range as reflected by the MUA. Furthermore, the MUA is systematically and inversely correlated with the amplitude of alpha-oscillations in the model (see Table 1).

**Table 1:** Correlation coefficients for the relationship between cortical alpha power and hemodynamic response of the individual nodes as well as the correlation of cortical alpha power and MUA across all nodes (CSF = 0.6).

	Mean correlation coefficient	Significance (p-value)	+/- confidence interval (95%)
Alpha vs. MUA cortex	-0.06	0.0012	0.03
Alpha vs. BOLD cortex	-0.25	8.6e-04	0.12
Alpha vs. MUA thalamus	-0.09	5.8e-05	0.03
Alpha vs. BOLD thalamus	-0.20	6.7e-04	0.09
Alpha vs. MUA reticular nucleus	-0.02	0.3019	0.03
Alpha vs. BOLD reticular nucleus	-0.17	0.0019	0.09

**Fig 5:**
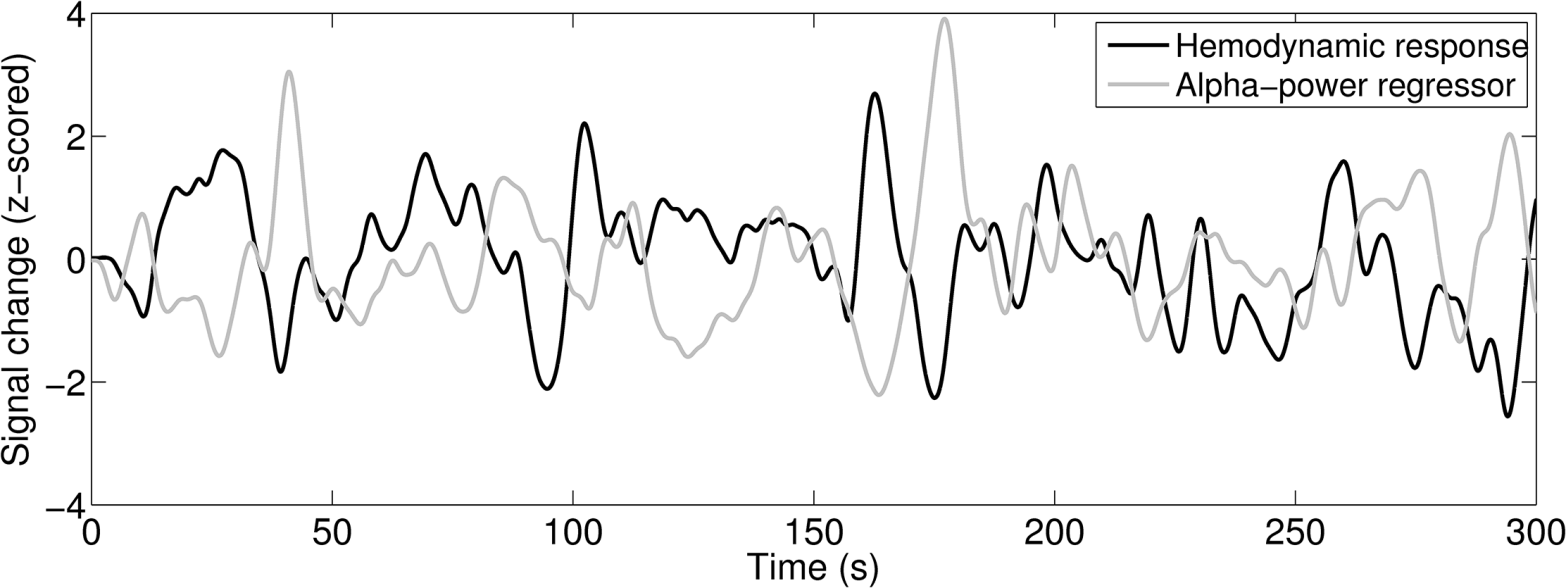
The ‘alpha BOLD regressor’ (grey line) derived by convolving the approximated alpha power time course with an HRF shows an inverse relationship to the approximated hemodynamic response of the cortical node (black line, CSF = 0.6) derived by convolution of weighted excitatory and inhibitory net neuronal activity and the HRF.

The resulting hemodynamic response of the model for the cortical node is depicted in Fig 5. Overlaid on this estimation is the predicted alpha power based on convolution of the time-frequency activity in the alpha-band originating from the cortical node. It results in an inverse relationship between the convolved alpha-power and the modelled hemodynamic response (correlation coefficients and statistics for all nodes can be found in Table 1).

### Relationship between alpha phase and firing rate

**Fig 6:**
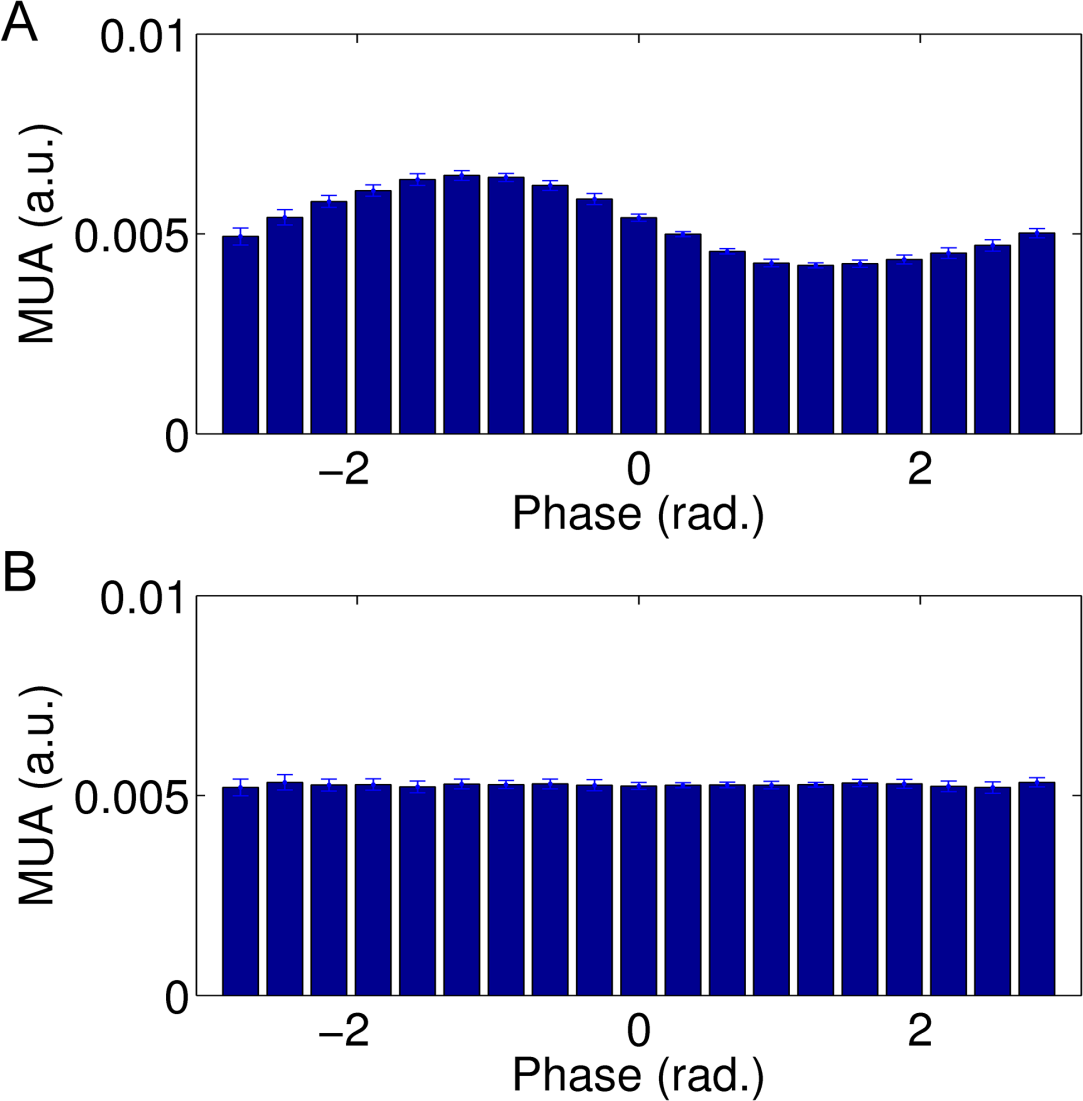
Alpha-phase dependency of the firing rate. Top panel shows alpha phase vs. MUA behaviour within the cortical node. Bottom panel shows a control condition: alpha phase in the cortical node vs. MUA behaviour from a disconnected node (CSF = 0.6).

Furthermore, the analysis regarding the relationship of alpha phase and MUA shows a clear dependency as visualized for the cortical node (Fig 6A). MUA is affected as a function of phase of the ongoing alpha activity. In contrast, if we relate the cortical alpha power to a disconnected yet alpha-like oscillating node, we observe no coupling of alpha phase and MUA (Fig 6B).

#### Variation of global connectivity–effect of connectivity scaling factor CSF vs. cortical spectral power and spectral coherence

**Fig 7:**
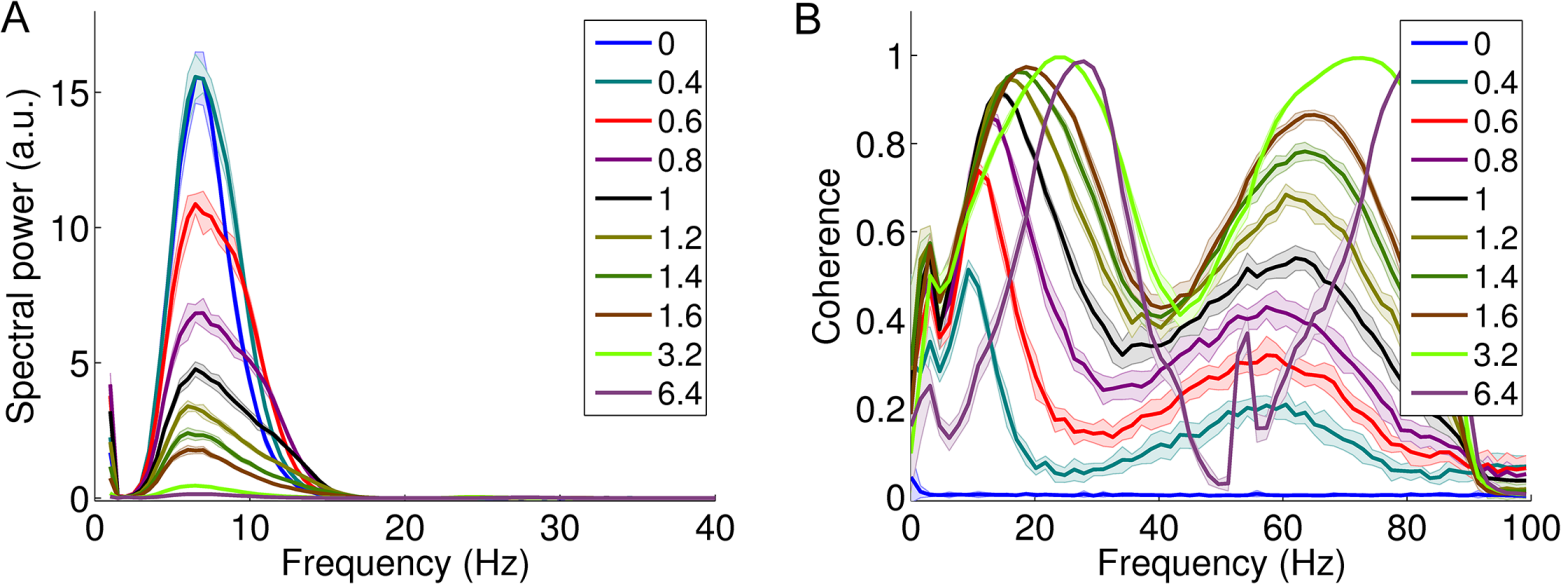
*A*. The effect of increasing global connectivity strength (CSF) on the LFP spectrum in the cortical node. In a highly linear fashion, spectral power in the alpha band drops to negligible levels with increasing CSF. ***B*.** The effect of increasing CSF on alpha-band coherence between thalamic and cortical node. The focus of coherent activity exhibits a shift to higher frequencies with increasing CSF. Thick lines represent average spectral power and coherence, while the shaded areas represent the standard deviation across repetitions for each level of CSF.

With increasing CSF, spectral power in the alpha frequency range around 10 Hz decreases (Fig 7A). In contrast to the decreasing mean spectral power, CSF correlates positively with spectral coherence in the alpha band (Fig 7B). Yet with increasing CSF, coherence also increases at higher frequencies–for example in the gamma range. Furthermore, the increasing CSF there leads to a shift from 10 Hz to 20 Hz as focus of the coherent activity, not paralleled by spectral power (Fig 7A).

#### CSF and alpha-MUA relationship

**Fig 8:**
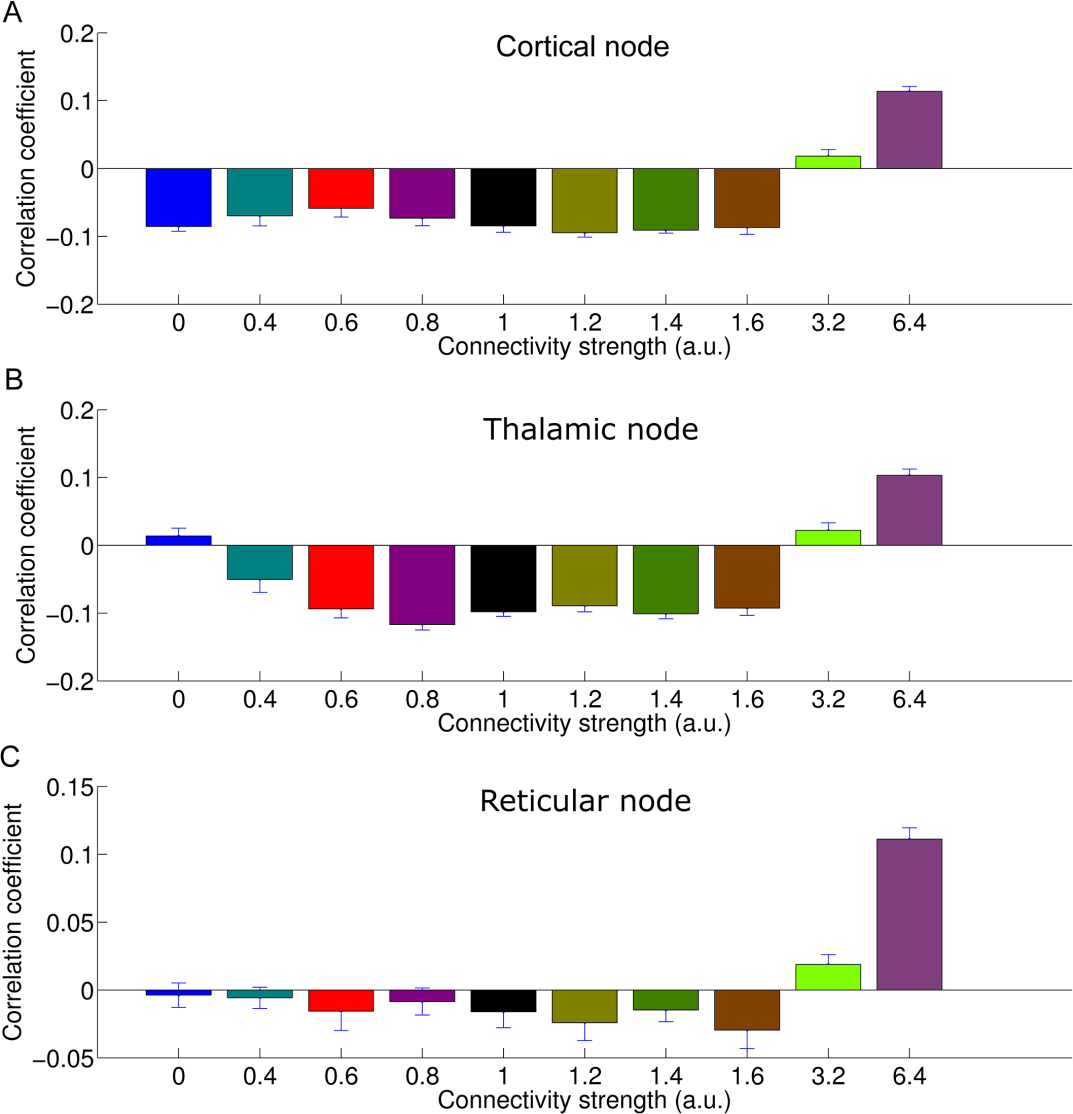
*A*. The effect of global connectivity strength (CSF) on the correlation between alpha-band power and firing rate. Effect in cortical node. ***B*.** Effect in thalamus. ***C*.** Effect in reticular nucleus. The inverse relationship remains stable across a broad range of connectivity levels and is not highly sensitive to the general power within the alpha band (which decreases with increasing CSF, see Fig 4). Error bars indicate standard deviation across repetitions. For most scenarios, apart from the hyper-excitation scenarios, we see a negative correlation between alpha-band power and firing rate as estimated by MUA.

For most of the range of CSF, amplitude of cortical alpha oscillations is inversely related to MUA in the three different nodes, i.e. reticular nucleus, thalamus and cortex (Fig 8A–8C). Only at the highest CSF value of 6.4 this relationship switches for all three nodes.

#### CSF and alpha—hemodynamic response relationship

**Fig 9:**
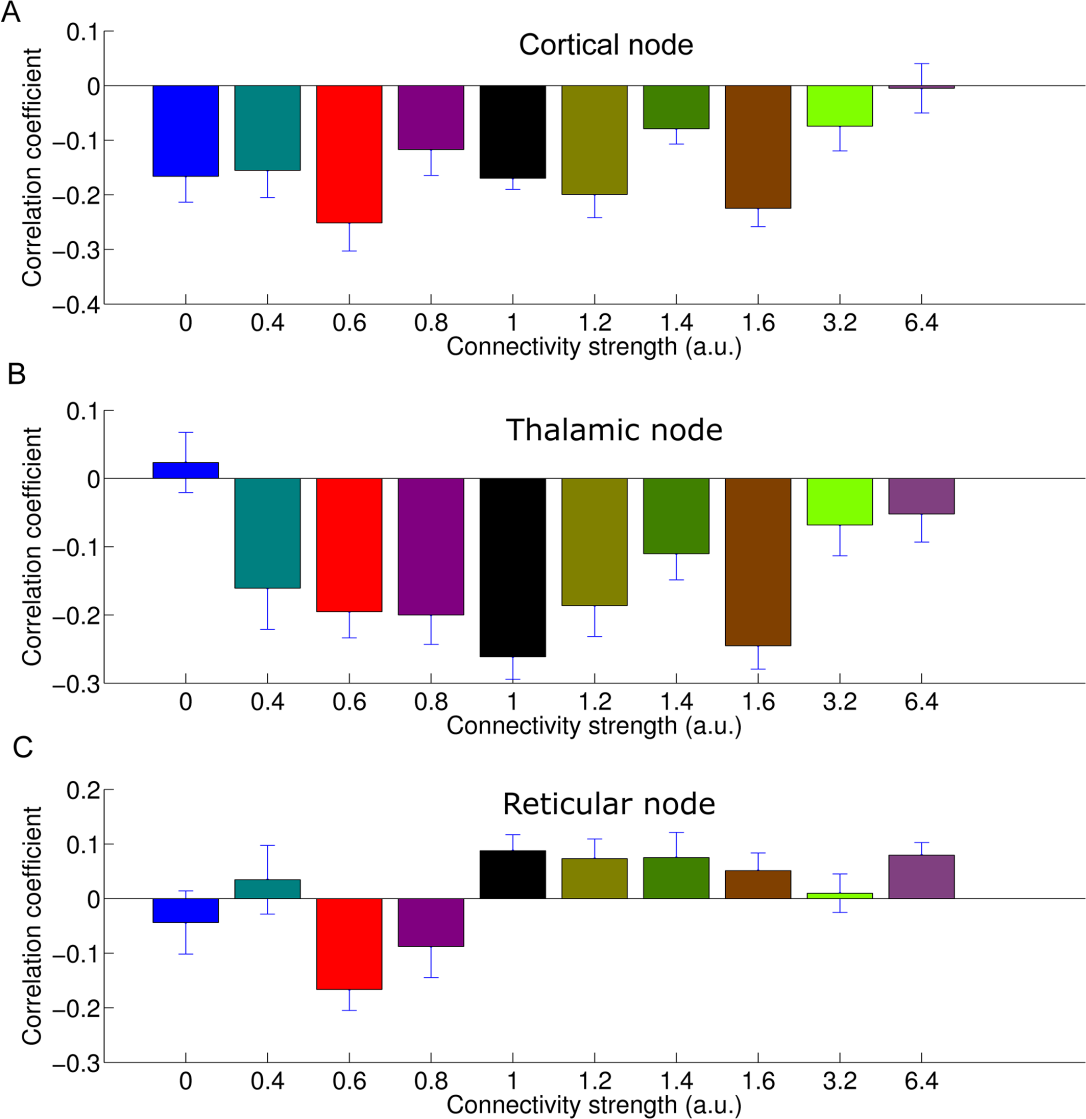
*A*. The effect of global connectivity (CSF) on the correlation between alpha-band power (convolved with HRF) and the predicted BOLD signal. Effect in cortical node. ***B*.** Effect in thalamus. ***C*.** Effect in reticular nucleus. This relationship remains inverse across the employed connectivity levels in cortical node and thalamus while it is less stable in the reticular nucleus. Error bars indicate standard deviation across repetitions.

Correspondingly, the hemodynamic response is also inversely related to the amplitude of cortical alpha oscillations. This behaviour is stable across the range of CSF levels for cortical and thalamic nodes (Fig 9). The strongest inverse correlation in the cortical node occurs for medium CSF and thus, medium average alpha power (at CSF = 0.6, Fig 9). At this CSF value all three nodes exhibit an inverse relation of alpha and the predicted BOLD activity. For the estimation of statistical significance, all analyses of the relationship of cortical alpha power to hemodynamic responses in the respective nodes are based on a sampling rate of 1 Hz while the correlation analysis of alpha power to firing rate was based on the original sampling rate of the model output.

## Discussion

Summarizing the results of our study, we have shown how a large-scale neuronal network model comprising coupled meso-scale region models can integrate empirical findings related to alpha oscillations. Using the burst-capable SJ3D neural-mass model of coupled brain areas, we provide a numerical simulation to account for the inverse relation between the alpha rhythm and the average firing rate behaviour of the underlying neuronal ensemble, which in turn is responsible for the hemodynamic response. In the subsequent discussion we focus on the following questions: What do our results imply and what can we infer from the model?

### The present modelling approach in the context of previous ones

There are numerous studies modelling the human alpha rhythm with varying degree of biophysical realism. For example, [15] developed an algebraic model with no direct link to its biophysical underpinnings, yet suitable for model inversion. Another class of models has more direct biophysical underpinnings. However, earlier studies often do not include the thalamus [4,5,16,17]. Some do include the thalamus, e.g. [18] or [7,19]–the latter modelling detailed nonlinear features of alpha activity such as bistability and scale invariance as reported in [20]. A few studies approximate the fMRI BOLD based on coupled neural mass models [16,21–24]. Some studies also try to make a theoretical conclusion about a possible relation between oscillations visible in EEG and metabolism and hemodynamics [25].

There has been relatively little theoretical work on the relationship between oscillations and haemodynamic responses. One theoretical heuristic provided in [26] builds upon earlier work examining the (necessary) relationship between fast oscillatory dynamics and mean activity levels in biologically realistic ensembles of coupled neurons [25]. In brief, BOLD signals reflect thermodynamic work and are proportional to neuronal firing rates. Empirically, a loss of alpha activity or desynchronization is usually associated with an increase in beta or gamma activity and reports an activated brain state and increased metabolic activity. In this work, we use a mean field model of neuronal dynamics (that captures some key dynamical behaviours) to verify this heuristic using neuronal simulations. What we add here with the present study is the potentially important role of bursting activity that may explain the coupling between multi unit activity and the phase of alpha firing. This coupling is another aspect of electrophysiological responses that can be measured empirically. Another relevant paper [27] proposes a mesoscopic dynamic equation system similar to the one employed here to explore the relations between EEG rhythms and BOLD signal.

While all those previous theoretical and numerical studies contributed considerably to our understanding of brain network activity, none of them focused on a possible link between a neuronal model capable of bursting behaviour and the empirical observations of an inverse relation between alpha and the BOLD signal. The present simulation reproduces these empirical findings and thus provides a first hint at the possible key physiological mechanisms.

### Modelling the translation from neuronal activity to the BOLD signal

We have assumed that the neurovascular drive behind the BOLD response is a weighted mixture of depolarizations in both inhibitory and excitatory sub-populations (e.g. see in [28]). This is an important assumption that may be contentious. It is generally thought that neurovascular coupling is largely driven by presynaptic glutamate release acting via glial cells and various endothelium relaxing factors. The biophysical mechanisms behind this coupling are reviewed in [27]. For the present purposes, we assume that large deviations in our state variables are sufficient to report or reflect this neuronal drive. However, in future work it may be possible to use empirical EEG and fMRI data to test various hypotheses about this coupling using dynamic causal modelling (e.g., [29]). In other words, use Bayesian model comparison to evaluate the precise variables that best predict BOLD responses while, at the same time, predicting local field and possibly MUA responses.

The relationship between alpha and specific state variables of the model is interesting in itself. However, the scope of this study was to reproduce the empirically observed relationship between alpha and BOLD. Multiple scenarios are conceivable which might lead to the empirically observed negative alpha vs. BOLD relationship, the here presented case is one scenario among others. Furthermore, it appears that due to low-pass filtering by the HRF kernel the correlation of alpha vs. BOLD gets enhanced in comparison to the observed alpha vs. firing rate relation in our simulation.

### The relation between alpha and BOLD in the thalamus

It might be worthwhile to note, that we did not reproduce the finding of a positive relationship between thalamic BOLD activity and cortical alpha activity as found in several empirical studies [8–11,30,31]. However, notably, there are other resting state studies which did not report such findings regarding the thalamus [32]. A similarly unclear situation exists with regard to positron-emission-tomography (PET) and alpha fluctuations. Some groups reported negative correlations [33,34], another reported a positive relation [35]. Also we would like to point out that this relation seems to be modulated by the condition eyes closed versus eyes open. For example, while a positive correlation between cortical alpha and thalamic BOLD was reported in the eyes closed condition (Moosmann et al. 2003), we found a negative relationship in an eyes open task-related condition [36]. Regarding the present modelling study, it is also important to note that, while we fail to generate a positive relationship in thalamic areas, in simulated data we do reveal a less negative relationship between cortical alpha and thalamus BOLD when compared to the relationship of cortical alpha vs. cortical BOLD. Alpha vs. MUA correlation in reticular nucleus is weakly positive, yet fails to reach significance. This is in contrast to the result for the cortical node. This might be a guide to future studies where to look for the relation between ξ and α state variables. Another important addition for future studies might be the differentiation between nuclei of the lateral thalamus such as pulvinar and lateral geniculate since empirical evidence indicates different functional roles for alpha rhythm generation.

### Oscillations, coherence and firing rates

The present thalamo-cortical model yields pronounced oscillations with underlying neuronal features such as coherence between regions or average firing rate behaviour conforming to empirical data from animals and humans. Spontaneous fluctuations of neuronal activity as visible in EEG are based on the fact that spontaneous neuronal activity is highly structured in space and time [37–39]. The synchronization of activity within neuronal populations gives rise to large (summed up) potential fluctuations that are visible in the EEG [40]. It is also known that these spontaneous synchronous LFP fluctuations are linked to the MUA of the respective neuronal population [39].

Our models shows good concordance with existing studies in that it expresses an oscillation of which the amplitude is negatively correlated with a) the firing rate and b) the approximated hemodynamic signal. This at first sight counter-intuitive behaviour is rendered reasonable when considering a firing-rate behaviour dependent on alpha rhythm phase. Similar to what has observed empirically, e.g. in [6], there is a stronger link between alpha rhythm *phase* and MUA than between alpha rhythm *amplitude* and MUA. Still, on average and, in our model, quite consistent across a wide range of connectivity scenarios, firing rate goes down with higher alpha amplitude.

An additional interesting finding is that in the range of our tested scenarios, we can identify one (CSF = 0.6) which shows–analogue to empirical observations–high coherence between regions on the one hand and local inverse correlations between alpha amplitude and firing rate on the other.

### Role of the network and the thalamus

We here explored the role of the large-scale network included in the model. More specifically, we tested if considering just a single node yields similar relations between alpha oscillations and neuronal firing as well as the hemodynamic response. We systematically explored the emerging activity in a network consisting of thalamic and cortical nodes. A detailed description of dynamics in a thalamo-cortical network model has also been provided by [41,42]; yet with a focus on fast oscillations in the 20 to 60 Hz range.

While for a disconnected model (CSF = 0.0) we already can reproduce some features of the alpha rhythm such as the inverse relationship between firing rate and alpha power, this does not imply that it is a realistic condition. Increasing connectivity between nodes has effects on other features of our network model such as cross-nodal coherence. Several models generate rhythmic behaviour through the use of transmission delays between coupled populations of spiking neurons. In isolation these populations do not show oscillations. Using the SJ3D model is different in that respect, since here, the single nodes are able to oscillate on their own. This scenario appears to be supported by empirical findings of reports of alpha activity in dogs where a distinct, but not perfect coherence between thalamic and cortical structures has been found [2]. However, even after ‘virtually’ eliminating the pace-making and driving effect of the thalamus by computing the partial coherence for the cortical electrode sites, the authors could show that intracortical coherence in the alpha band was still high. This was taken as indication that the thalamus is not the only structure expressing rhythmic activity (which then is transmitted), but that the cortical areas might oscillate on their own. This holds also true in our case, since ‘deafferenting’ a node by decoupling it from the others might still yield an oscillating node. However, temporal dynamics of these oscillations are then governed by local interactions and not more distant interactions.

In summary, just a single node can reproduce the observed negative correlation between cortical alpha and firing rate/ BOLD. Interestingly, this negative relationship between alpha and BOLD in the cortical node is maintained even when including the thalamic and reticular nodes–which is not trivial. More importantly, however, here we were looking for a model, which can reproduce multiple phenomena, with one important feature being the observed coherence between thalamic and cortical alpha oscillations. This can only be achieved by introducing connectivity between the thalamic and cortical node. In that sense, only our connected network demonstrates the reproduction of these features combined, which, in our view, makes this model more interesting without adding needless complexity. Future investigations will investigate whether the network shows additional features, which cannot be accomplished by modelling just a single node.

### Role of bursting

Specifically, we demonstrate how a cellular ensemble in bursting mode oscillates in the alpha frequency regime and how that translates into alpha phase-dependency of the underlying MUA. Furthermore, we also demonstrated how the bursting mode, i.e. the alpha oscillations, translate into lower average firing rate of the involved neuronal ensemble. It is important to note that bursting per-se does not simply explain the observed average inverse behaviour of alpha amplitude and MUA. Bursting behaviour is characterized by the mutual presence of two time scales, a fast spiking and slower bursting time scale. These two time scales allow interactions between and across scales. For instance, it is significantly easier to achieve synchronization across neurons on the slower bursting time scale than on the faster scales [43]. Now why would bursting imply that alpha activity–i.e. the slower time scale–is inversely related to MUA activity–the faster time scale? In principle, a scenario is conceivable where bursting implies a *higher* average MUA than in the tonic mode. However, in most cases empirically observed, the bursting mode is associated with the local firing rate activity being generally lower than during non-bursting, which also holds true in case of our model. The activity within nodes goes into a bursting-like mode, when these nodes are less excited by incoming input. This fits well with the reports from monkey data as well as with numerous EEG-fMRI studies demonstrating less metabolic demand with higher alpha amplitudes.

### Integrating our findings in existing concepts

The ‘gating by inhibition’ theory [44] ascribes the alpha rhythm an inhibitory role mediated through the temporary suppression of gamma oscillations that in turn have been shown to be positively related to neuronal population firing and visual processing [45]. While this theory has prevailed for some time, the biophysical link between the decreased firing and increased alpha oscillations was missing. While we did not focus on gamma band activity specifically, the relationship between high-frequency neuronal activity and alpha power is clearly visible in our model. The firing rate dependency contingent on alpha phase in our model fits very well with this idea of cyclic inhibition. In our model, it is basically a local phenomenon, but due to the observed alpha coherence across nodes it might also play a role in the connected circuit.

### Insight from the model for the functional role of the alpha rhythm

The alpha rhythm is the most prominent oscillatory electric large-scale signature of the human brain. In previous empirical studies, we and others found converging evidence for spatially and functionally distinct alpha brain states affecting cognition, behaviour, learning as well as evoked brain responses [36,46–53]. The fact that there are spatially and functionally distinct patterns of alpha activity support the idea that the human brain holds various alpha rhythm sources. In addition to the most prominent ‘classical’ alpha rhythms that can be found predominantly over posterior brain regions, also other sensory systems are equipped with resting state alpha like oscillations, such as the tao rhythm in the auditory and the mu rhythm in the sensory-motor system. Similar to multimodal analyses of occipital alpha, spontaneous modulations of the power of the mu rhythm have been shown to exhibit a similar inverse relation with the BOLD signal in the underlying cortical regions [54] as observed for the classical alpha rhythm. This is of importance since it might point to a universal mechanism underlying the inverse relation between the cortical BOLD signal and alpha oscillations. Our model supports this notion since it is blind to which modality might be involved. It generalizes to any network, which is connected in a similar way, i.e. via a thalamic relay nucleus to the cerebral cortex and a modulating, inhibitory nucleus such as the reticular nucleus of the thalamus.

### Outlook

The model presented here is a computationally efficient yet powerful simulation of a thalamocortical circuit able to generate alpha-like rhythms with features close to what is empirically observed in human and animal brain oscillations in the alpha frequency range. We believe that this model shows remarkable promise and might be extended to capture additional features of spontaneous human brain activity. It will be interesting to embed this specific network in a more global network on a whole brain level (see for example [22] for a full brain model based on SJ3D nodes or [55] for a full brain spiking neuron model) in subsequent studies. As an extension to [22] we would add inhibitory connections and node-specific intrinsic connectivity configurations such as modelled here for the reticular nucleus. That being said, the model described here already generates useful insights on how the alpha rhythm relates to neuronal firing and the BOLD signal, it offers new hypotheses for future work, and points to an important role of bursting behaviour for large-scale EEG dynamics.

## Methods

To study how neuronal oscillations and their concurrent firing rate as well as hemodynamic response relate to each other, we employed a model of neuronal dynamics coupled through a thalamo-cortical network. The neural mass model, used to describe the dynamics at the network nodes, was the Stefanescu-Jirsa population model. This neural mass model comes in two flavours, the Stefanescu-Jirsa 2D model composed of FitzHugh-Nagumo neurons and the Stefanescu-Jirsa 3D model composed of Hindmarsh-Rose-neurons [13] and can be found as download packages at http://www.thevirtualbrain.org/tvb/ [56] after registration, corresponding source code under https://github.com/the-virtual-brain/tvb-library. The authors used techniques derived from nonlinear system theory [57], in which coupled neurons with parameter dispersion (for instance distributed firing thresholds) reorganize themselves into clusters displaying similar dynamics. Due to the clustering in state space, traditional mean field techniques fail, but a decomposition of the total population dynamics into different activity regimes called “modes” allows for a reconstruction of the dynamic features of the population. Mode decomposition techniques are frequently used in physics and engineering offering solutions for a variety of problems [13]. Here each mode effectively tracks one of the clusters of neurons in state space and it has been shown that 3 modes suffice to follow closely the dynamics of the complete neuronal system containing hundreds of neurons. Importantly, single neurons may switch from one cluster to another and thus change their firing pattern. This underwrites the relevance of the population modes, which capture the emergent behaviours of the population, independent of microscopic details such as neuronal membership. The derivation of a new set of differential equations describing the evolution of these modes makes it possible to capture the complex dynamic repertoire of a total population via a closed set of self-consistent neural mass equations (for details please see [13]). The original Hindmarsh-Rose (HR) model was developed to reflect a wide range of neuronal activity and is capable of modelling the membrane potentials of neurons even during spike-bursting behaviour [14]. This individual neuron model comprises three coupled differential equations (see Eq 1). The different neuronal regimes of the HR neuron are demonstrated in Fig 1.

$$\begin{array}{l} \dot{x}=y-ax^{3}+bx^{2}+I-z\\ \dot{y}=c-dx^{2}-y\\ \dot{z}=r(s(x-x_{1})-z) \end{array}$$

**Equation 1.** The original Hindmarsh-Rose equation for modelling single neurons. Depending on parameter *I*, which regulates general excitability of the respective model neuron, this system may exhibit a variety of behaviours (e.g. see Fig 1). The three state variables are x, y and z, where, x and y are varying on a faster time scale, while z varies on a slower scale (*r*<0.001). Given commonly used values for the parameters *a*, *b*, *c*, *r*, *s* are found in [14] and Table 2.

**Table 2:** State variables and parameters of the model and corresponding values used in the present study.

Parameter	Value	Description of parameters
a, b, c, d	1, 3, 1, 5	Constants affecting faster ion channels
r	0.006	Constant affecting slower ion channels
s	4	Bursting strength of model
μ and σ	3.1, 0.5	Mean and dispersion of input current in each node
IE, II	Derived from μ and σ	Models excitability of each node and mode (IE for excitatory input, II for inhibitory input)
CSF	0.0–6.4	Coupling scaling factor for connections between nodes
Inverse speed	1/7	Scales delay for defined internode distances
Internode distances		
TC	8.8	Thalamo-cortical distance
TR	0	Thalamo-reticular distance
Intrinsic connectivity (within nodes)*		
K11	0.5	Excitatory—excitatory coupling
K21	0.5	Inhibitory-Excitatory coupling
K12	0.25	Excitatory-Inhibitory coupling
*Extrinsic connectivity (between nodes)*		
TC	2	Thalamo-cortical coupling
TR	0.5	Thalamo-reticular coupling
CT	1	Cortico-thalamic coupling
CR	1	Cortico-reticular coupling
RT	-2	Reticulo-thalamic coupling
*State variables*		
*ξ*		Membrane potential of EPs
*η*		Spiking variable of EPs
*τ*		Slow bursting variable of EPs
*α*, *β*, *γ*		Corresponding values for IPs

EPs = Excitatory (sub-)population, IP = Inhibitory (sub-)population

* with the exception of reticular nucleus which accommodates only inhibitory neurons and thus no intrinsic connectivity [3].

Depending on the input parameter I, which can be interpreted as an external input or the degree of membrane excitability, this model shows a wide range of neuronal behaviour from fixed point dynamics through spike-burst and chaotic behaviour to regular self-sustained oscillations (Fig 1). However, even when utilising this simplified approach, modelling every single neuron in larger cell assemblies implies considerable computational load. Thus, a further reduction of the number of units to model is desirable. For this, neural mass modelling techniques are an attractive solution [57], especially when they have been shown to reproduce dynamics of non-reduced neuronal population models as the specific realization employed here [13,58]. We placed the neural mass models at each network node interconnected via a circuit comprising a lateral thalamic node, a cortical node and (the thalamic) reticular nucleus. This is depicted schematically in Fig 2A. The network's structure included a reciprocal excitatory connection between thalamic and cortical node neurons, as well as an excitatory thalamo-reticular connection. The reticular nucleus in turn exerts inhibition on the thalamic (relay nucleus) node. The chosen connectivity scheme is in accordance with what is known about typical thalamo-cortical circuits [3]. Also, the Euclidean distance between these nodes, in combination with an assumed conduction speed (see Table 2), was used to account for the expected delays in interaction.

Each node of the thalamo-cortical model consists of an excitatory neuron population and an inhibitory interneuron population, except the reticular nucleus which, according to literature, is modelled with inhibitory neurons only [59]. The activity of the individual uncoupled nodes is described by the following equations:

$$\begin{array}{l} {\dot{\xi }}_{i}=f(\xi_{i},\eta_{i},\tau_{i},\alpha_{k})=\eta_{i}-a\xi_{i}{}^{3}+b\xi_{i}{}^{2}-\tau_{i}-K_{11}\left|\sum_{k=1}^{3}A_{ik}\xi_{k}-\xi_{i}\right|-K_{12}\left|\sum_{k=1}^{3}B_{ik}\alpha_{k}-\xi_{i}\right|+IE_{i}\\ {\dot{\eta }}_{i}=c_{i}-d_{i}\xi^{2}{}_{i}-\eta_{i}\\ {\dot{\tau }}_{i}=rs\xi_{i}-r\tau_{i}-m_{i}\\ {\dot{\alpha }}_{i}=\beta_{i}-e_{i}\alpha^{3}{}_{i}+f_{i}\alpha^{2}{}_{i}-\gamma_{i}+K_{21}\left[\sum_{k=1}^{3}C_{ik}\xi_{k}-\alpha_{i}\right]+II_{i}\\ {\dot{\beta }}_{i}=h_{i}-p_{i}\alpha^{2}{}_{i}-\beta_{i}\\ {\dot{\gamma }}_{i}=rs\alpha_{i}-r\gamma_{i}-\eta_{i} \end{array}$$

**Equation 2.** The Stefanescu-Jirsa 3D model describes the dynamics of a population of coupled excitatory and inhibitory Hindmarsh-Rose neurons, which serves as a network node of the larger network. The excitatory population dynamics is described by three modes, of which each, analogous to Eq 1, comprises three state variables, *ξ*_*i*_, *η*_*i*_ and *τ*_*i*_, where the index *i* denotes the *i-*th mode and *a*, *b*, *c*, *d*, *r*, *s* are constant parameters. In full equivalence, the inhibitory population comprises three state variables *α*_*i*_, *β*_*i*_, *γ*_*i*_ of the *i*-th mode and *e*, *f*, *h* are constant parameters. *A*_*ik*_, *B*_*ik*_ and *C*_*ik*_ are coupling constants, which can be derived analytically from the fully microscopic neuron system and can be found in Stefanescu & Jirsa (2008, cf. Supplementary Text S1).

*ξ*, *η*, *τ* represent state variables of the excitatory sub-population whereas *α*, *β*, *γ* represent state variables of the inhibitory sub-population. Their modes (indexed by i) are directly related to the distributions of the dispersed threshold parameter. In its original single-neuron formulation—that is known for its good reproduction of burst and spike activity and other empirically observed patterns—the variable *ξ(t)* encodes the neuron membrane potential at time t, while *η(t)* and *τ(t)* account for the transport of ions across the membrane through ion channels. The spiking variable *η(t)* accounts for the flux of sodium and potassium through fast channels, while *τ(t)*, called bursting variable, accounts for the inward current through slow ion channels [14]. The parameters of the neural mass equations are directly related to the biophysical parameters and their dispersion through the mean field averaging performed [see supplementary materials in [13] for explicit formulae]. If the resulting “modes” (capturing the distributions of the dispersion) are orthogonal, then the equations for the neural mode dynamics are formally equivalent to the single neuron dynamics except for the coupling. Orthogonality is defined by non-overlapping rectangular functions, which represent the threshold distributions of membrane excitability. Each of the resulting three modes of the SJ3D model reflects distinct dynamical behaviours. For example if uncoupled, the modes capturing the lower values of membrane excitability would account for the neurons, which mostly maintain sub-threshold behaviour while the modes capturing higher values of excitability would then account for neurons with periodic discharges. All parameters of the SJ3D nodes were derived from the HR single neuron model parameters (Table 2). Parameter I reflects the general excitability of a single neuron and contributes to the overall excitability of a particular mode. In our case, this parameter is split up into two components: IE is the input (current) to the excitatory subpopulation (EP) of each node; II is part of the input to the inhibitory population (IP) of each node. Its function is analogous to the function of I in the original formulation of the single neuron model [14]. These values are derived from the parameters μ and σ (see Table 2), which for each node govern the mean level and dispersion of excitability across all modelled subpopulations. We chose the parameter μ (the mean) and σ (the dispersion) such that the resulting system expresses sufficient spontaneous bursting behaviour but is neither completely chaotic nor in a rigid limit cycle, nor attracted to a fix point. Other parameters specific to the Stefanescu-Jirsa 3D model (see Eq 2) are the following: K11, K12 and K21. These define the intrinsic connectivity between EPs and IPs (for the exact parameter values used in the present study see Table 2). The same values are implemented for all nodes with the exception of the reticular nucleus, which is known to contain almost exclusively inhibitory neurons [3] and thus, it is modelled with inhibitory neurons only. In general, the presented model embraces inhibition as an important neuronal mechanism in two ways. First, there is the inhibition between nodes, such as that performed by the reticular nucleus, which is implemented by a negative, i.e. inhibitory extrinsic coupling of reticular nucleus and thalamus (Fig 2A). Second, within each node and mode, there is a population of inhibitory interneurons coupled to the excitatory population. A visualization of this intrinsic coupling is provided in Fig 2B. Besides this intrinsic connectivity, extrinsic connectivity will crucially determine the outcome of the network dynamics. The resulting evolution equation is displayed in Eq 3:

$${\dot{\xi }}_{i}(t)=f(\xi_{i},\eta_{i},\tau_{i},\alpha_{k})+c\sum_{j=1}^{N}w_{ij}\xi_{j}(t-\Delta t_{ij})+\varepsilon (t)$$

**Equation 3.** The activity of each coupled network node is now composed of its own intrinsic dynamics *f(ξ*_*i*_,*η*_*i*,_ *τ*_*i*_,*α*_*k*_ *)* (as generated by the six equations in Eq 2) and coupled to the other nodes via the coupling with strength *w*_*ij*_, the time delay (resulting from the distance between individual nodes divided by the assumed conduction velocity, see Table 2.) and a constant scaling factor c. Noise is added by term *ε(t)*. To drive the system, noise was inserted into each node. The noise level was chosen to not saturate the system, while providing sufficient dynamic behaviour. The noise source was white with Gaussian amplitude (mean = 0, standard deviation = 0.1). Numerical integration of the system was performed using Heun’s method [60], with an integration step size of 0.05 ms. Each run comprised a time window of 300s. All simulations were run on a Matlab-based version (R2014a, Mathworks, Natick, USA) of the above mentioned TVB toolbox packages.

### Data simulation using a coupled network of the SJ3D nodes

The SJ3D model provides three distinct ‘modes’, which reflect the major degree of variation of neuronal activity within a neuronal mass (see Eq 2 and [13]). For each mode and node, the relevant state-variables are ξ and α, representing the summed membrane potentials of the respective excitatory and inhibitory sub-populations. We down-sampled data from its original 20kHz to 2kHz for all subsequent analyses. We examined the output of the system in a two-fold manner: One type of output is based on the high-frequency (500–900 Hz) part of the resulting membrane potential in analogy to multi-unit activity as measured in real data, e.g. as analysed in [61], [62]. This output we refer to as an approximation of multi-unit activity (MUA). Another type of output was generated by extracting the low-frequency (5-60Hz) part of the signal in analogy to the analysis of local field potentials (LFP). The resulting signal is referred to as an approximated LFP. Data was filtered with a 2nd order Butterworth filter. For both approximated LFP and MUA, the excitatory activity of all three modes (state variables *ξ*, *η*, *τ*) was summed to reflect the total activity of both measures within each node. Inhibitory modes (*α*, *β*, *γ*, see Eq 2) were ignored in this approximated activity, as it is the excitatory pyramidal neurons that provide the dominant contribution to the EEG and the alpha rhythm (e.g. [63]).

### Fluctuation of alpha amplitude, MUA and their mutual relationship

To estimate the oscillatory activity of the cortical node in the alpha-band, we performed time-frequency decomposition by wavelet decomposition of the resulting LFP using a Morlet mother wavelet (3 cycles) and used its modulus to extract the time evolution of the average power within the frequency band of 8–12 Hz.

The mesoscopic model does not provide direct access to single cell activity and spiking. Hence we approximated the local firing rate within each node by calculating the power envelope across the entire chosen frequency band of 500–900 Hz for the approximated MUA output. To obtain a measure of how the cortical alpha power relates to MUA in the individual nodes of our model, we smoothed the MUA power time course (sliding average, with 75 samples, (i.e. 375 ms) lead and lag, resp.). Subsequently we performed a linear correlation analysis of the resulting alpha-band power of the cortical node and the resulting smoothed MUA power.

### Prediction of the hemodynamic response and the ‘alpha BOLD regressor’

We predicted the hemodynamic response of each node by convolving the direct output of the excitatory as well as the inhibitory membrane potentials for each mode (i.e. state variables *ξ* and *α*) with the canonical hemodynamic response function (HRF) characterized by a peak response at 5 s as used by the statistical parametric mapping software SPM (Functional Imaging Laboratory, London, UK). This function closely resembles the results generated with the biophysically based Balloon-Windkessel model [64]. Then, the resulting signal time courses were summed up within each mode with the respective weighting, i.e. inhibitory component 1/3 vs. excitatory component 2/3, and across all modes to obtain a final estimate of metabolic demand for each node [13].

Following the typical routines of empirical EEG-fMRI analyses—we convolved the time-course of the simulated cortical alpha power with the canonical HRF. This provided the ‘alpha BOLD regressor’. In a next step we performed a linear correlation analysis between the ‘alpha BOLD regressor’ and the predicted fMRI response linked to the net neural activity at the cortical node. After having performed these calculations for each node, we now determined the relation of the simulated cortical fMRI signal and the simulated and HRF convolved alpha power by analysing their linear correlation. The resulting correlation strength between the two simulated signals was then qualitatively compared to empirical observations in real data taken from our previous EEG-fMRI studies [11,36].

### Spectral coherence

We calculated the magnitude-squared coherence between the LFP from the cortical and thalamic node. The coherence indicates how well a band-specific signal corresponds to another signal in the same frequency band. It is a function of the spectral densities of these two signals and theirs cross-spectral density. For analysis, we used the cortical and the thalamic node. A value of 0 indicates absence of coherent frequency-band specific activity. A value of 1 implies that frequency-band specific amplitude fluctuations are perfectly in phase.

### Linking alpha phase and MUA

Approximated cortical alpha was sorted into 20 phase bins using the phase information from the wavelet analysis. For each bin MUA activity was estimated separately and averaged across repetitions (n = 10). This number was chosen to have a first approximation of variation of our observed effects. Phase dependency can be inferred from the resulting distribution of average MUA. A uniform distribution indicates no phase-dependency, while a sinusoidal distribution of MUA across bins indicates phase dependency. We performed this analysis for the cortical node and as a control condition across two disconnected nodes, i.e. phase of alpha oscillations of one node against MUA of another disconnected node.

### Effect of modulating global connectivity strength

We examined the effect of global connectivity strength on the described analysed features of the alpha rhythm. Specifically we determined the effects on spectral power, spectral coherence, the alpha-MUA relationship and the alpha-fMRI coupling. To this end, we modulated the parameter CSF, ranging from 0.0 (entirely disconnected model) to 12.8 (extremely strong coupled model). While the model showed saturation effects at CSF = 6.4, at the next level, at CSF = 12.8 the model started to collapse completely (due to hyper-excitation), so we did not use CSFs higher than 6.4. For each range of CSF the model was repeated 10 times (with randomized initial conditions) to obtain an approximation of the robustness of the observed effects. We focused on cortical alpha oscillations and their relationship to features of the underlying neuronal ensemble or its relationship with features of other nodes.
